# Indirect regulation of topsoil nutrient cycling by groundwater depth: impacts on sand-fixing vegetation and rhizosphere bacterial communities

**DOI:** 10.3389/fmicb.2023.1285922

**Published:** 2023-12-05

**Authors:** Lianyi Hao, Xiuhua Liu, Ruiqing Ji, Yandong Ma, Puxia Wu, Qingxi Cao, Yunling Xin

**Affiliations:** ^1^School of Water and Environment, Chang’an University, Xi’an, China; ^2^Key Laboratory of Subsurface Hydrology and Ecological Effect in Arid Region of Ministry of Education, Chang’an University, Xi’an, China; ^3^Key Laboratory of Eco-hydrology and Water Security in Arid and Semi-arid Region of Ministry of Water Resources, Chang’an University, Xi’an, China; ^4^Key Laboratory of State Forest Administration on Soil and Water Conservation & Ecological Restoration of Loess Plateau, Shaanxi Academy of Forestry, Xi’an, China

**Keywords:** groundwater table depth, plant diversity, rhizosphere functional bacteria, soil nutrient, sand-fixing plant

## Abstract

**Introduction:**

The impact of groundwater table depth (GTD) on bacterial communities and soil nutrition in revegetated areas remains unclear.

**Methods:**

We investigated the impacts of plant growth and soil physicochemical factors on rhizosphere bacterial communities under different GTD.

**Results:**

The four plant growth indices (Pielou, Margalef, Simpson, and Shannon-Wiener indices) and soil water content (SWC) at the Artem and Salix sites all showed a decreasing trend with increasing GTD. Salix had a higher nutrient content than Artem. The response of plant rhizosphere bacterial communities to GTD changes were as follows. Rhizosphere bacteria at the Artem and Salix sites exhibited higher relative abundance and alpha diversity in SW (GTD < 5 m) compared than in DW (GTD > 5 m). Functional microbial predictions indicated that the rhizosphere bacterial communities of *Artem* and *Salix* promoted carbon metabolism in the SW. In contrast, Artem facilitated nitrogen cycling, whereas Salix enhanced both nitrogen cycling and phototrophic metabolism in the DW.

**Discussion:**

Mantel test analysis revealed that in the SW of Artem sites, SWC primarily governed the diversity of rhizosphere and functional bacteria involved in the nitrogen cycle by affecting plant growth. In DW, functional bacteria increase soil organic carbon (SOC) to meet nutrient demands. However, higher carbon and nitrogen availability in the rhizosphere soil was observed in the SW of the Salix sites, whereas in DW, carbon nutrient availability correlated with keystone bacteria, and changes in nitrogen content could be attributed to nitrogen mineralization. This indicates that fluctuations in the groundwater table play a role in regulating microbes and the distribution of soil carbon and nitrogen nutrients in arid environments.

## 1 Introduction

In recent years, irrational human activities, including water diversion for irrigation and overexploitation, coupled with the impacts of climate change, have resulted in significant groundwater depletion. This phenomenon poses a severe threat to vegetation survival in arid and semi-arid regions worldwide (Castle et al., 2014; Barbeta et al., 2015). Groundwater is a crucial water resource in these drylands and is tightly linked to vegetation productivity and the ecosystem carrying capacity (Karthe, 2018; Ma et al., 2022). Alterations in the groundwater table, whether upward or downward, exert a substantial influence on the soil water content, consequently affecting regional vegetation growth (Chen et al., 2016). Vegetation growth predominantly relies on water within the capillary zone above the groundwater surface (Barron-Gafford et al., 2021). Numerous studies have demonstrated that drought disrupts vital ecosystem processes, pushing the sustainable application of water resources close to vegetation growth limitations, particularly in revegetated areas (Han et al., 2020; Li et al., 2021). Although vegetation restoration plays a pivotal role in rehabilitating degraded ecosystems, unregulated large-scale expansion of vegetation can disrupt the overall water balance in a region, potentially leading to soil fertility loss and detrimental alterations to the soil microbial structure. This can affect the progress of ecological restoration (Wall et al., 2004; Schlaepfer et al., 2017; Jia et al., 2020).

Numerous studies have demonstrated that a decrease in the groundwater table induces alterations in plant communities, modifies soil physicochemical attributes, and affects soil nutrient cycling, such as carbon and nitrogen dynamics (Liu et al., 2018; Zeng J. et al., 2020; Shen et al., 2022). Xi et al. (2018) established a robust association between the groundwater table and plant growth, particularly root development and distribution, emphasizing that GTD is a key determinant of rooting depth under relatively stable conditions. Mao et al. (2018) indicated the positive impact of groundwater table on both aboveground and belowground plant traits, along with a concurrent reduction in soil moisture content with decreasing groundwater table in the scope of shallow groundwater table depth. Fluctuations in the groundwater table exert diverse effects on soil physicochemical properties and environmental factors such as oxygen content (O2) (Meyerhof et al., 2016), temperature (Lai et al., 2021), humidity, atmospheric pressure and chemical composition (Shen et al., 2022). Vegetation and inter-root microorganisms are sensitive to environmental changes, allowing for rapid adaptation (Bissett et al., 2010; Hill et al., 2015). Therefore, variations in the groundwater table will inevitably affect the composition of inter-root microbial communities, resulting in distinct assemblages adapted to shifting groundwater conditions.

Microbial communities in the rhizosphere exhibited distinct characteristics among the different vegetation types. To some extent, changes in rhizosphere soil physicochemical properties are influenced by root regeneration and rhizosphere metabolism (Galland et al., 2019). To rehabilitate soil in the Mu Us Sandy Land, shrubs such as Artem and Salix have been extensively introduced. Artemisia ordosica has been reported to release allelochemicals through rain leaching, inhibiting the growth of other plants and potentially establishing a dominant community (Wang et al., 2022). In addition, *Salix* litter significantly increased soil organic carbon (SOC), total nitrogen (TN), and soil water content (SWC) (Li T. et al., 2019). The involvement of microorganisms is vital for litter decomposition and influences nutrient cycling within the ecosystem (Osburn et al., 2022). Rhizosphere microbes play a critical role in nutrient cycles, particularly in soil carbon and nitrogen dynamics, as well as in the conversion of both organic and inorganic matter, especially rhizosphere functional microorganisms (Zhao et al., 2020; Kunkun et al., 2022). Functional microorganisms, including photoheterotrophic, chemotrophic, and those involved in the degradation of aromatic compounds and methane generation, govern the decomposition and oxidative synthesis of organic carbon under varying conditions (Liang et al., 2019; Huang et al., 2020). Nitrogen fixation, nitrification, and nitrate reduction are the primary microorganisms responsible for regulating the nitrogen cycle (Chen et al., 2020; Xiang et al., 2020). Plant root secretions, including organic acids, affect microbial species and activity (Zhao et al., 2020). Nonetheless, the effects of the application of *Artem* and *Salix* in sandy soil restoration on properties such as SOC, TN, SWC, and TP, as well as soil microbial composition, remain unclear. Addressing this knowledge gap requires a systematic assessment of how associations between plant species and soil properties, including microbial diversity, evolve during the revegetation process.

The Mu Us Sandy Land, located in the arid and semi-arid regions of northwestern China, has encountered substantial ecological and environmental challenges to vegetation restoration due to drought and soil impoverishment (Veldkornet et al., 2016). Effective vegetation restoration measures have emerged as a pivotal strategy for conserving soil and water resources (Wu and Ci, 2002). However, certain areas have witnessed deterioration in plantation forest health (Bi et al., 2021). Since the implementation of the vegetation restoration program in 1999, rapid vegetation growth has induced notable fluctuations in the local groundwater cycle, potentially affecting the regional groundwater table (Jia et al., 2020). Furthermore, these alterations in the groundwater table can probably affect vegetation growth and nutrient cycling (Han et al., 2020; Jiakun et al., 2023), posing a serious challenge in the battle against secondary desertification in Mu Us Sandy Land.

This study utilized two common sand-fixing vegetation types, Salix and Artem sites, to examine changes in plant community composition, soil physicochemical factors, and rhizosphere bacterial communities across varying GTD. The hypotheses were as follows. In both the SW and DW sites, (1) it was postulated that significant variations would be evident in the characteristics of plants, soil physicochemical factors, composition of rhizosphere bacterial communities, functional bacteria, and co-occurrence networks of sand-fixing vegetation. (2) an extension of the hypothesis suggested that the impact of plant diversity and soil physicochemical factors on soil bacterial communities and functional bacteria would display noteworthy distinctions between the two sites. (3) it was conjectured that the regulation of soil nutrient levels might be contingent on differing levels of vegetation bacterial diversity and the presence of specific functional bacteria within these ecosystems.

## 2 Materials and methods

### 2.1 Study sites

**Figure 1:**
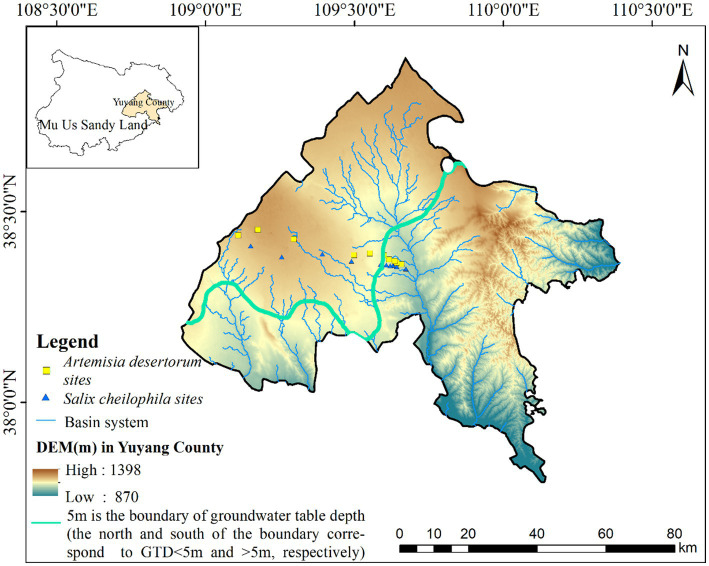
Distribution of plant sampling sites in Mu Us Sandy Land.

The study area, spanning approximately 259 km^2^, is situated on the southeastern fringe of the Mu Us Sandy Land (109.13°–109.72°E, 38.32°–38.45°N) at the transitional boundary between the Mu Us Sandy Land and the Loess Plateau. This region has a unique geographical context and hosts an exceptionally sensitive and delicate ecological environment (Figure 1). The average annual temperature ranges from 8.15°C to 10°C, with peak temperature typically occurring in July. The average annual precipitation ranges from 137 mm to 578 mm, gradually decreasing toward the northwest. Precipitation exhibits substantial annual variability, with 60–75% concentrated from July to September. Evaporation is intense, averaging 1,606–3,240 mm annually (Zhao et al., 2021). The total annual radiation ranges from 5,140 MJ/m^2^ to 6,281 MJ/m^2^, ensuring ample sunshine availability.

The study area exhibits complex and undulating terrain with a gradual descent from northwest to southeast. The groundwater table displays significant spatial variation, ranging from 0 to 60 m (Rao et al., 2022). In this region, the limit of evaporation depth for the groundwater table generally falls within the range of 4–6 m. For our delineation between SW and DW, we adopted a groundwater depth of 5 m based on distinct differences in soil water content and vegetation coverage, as established in our previous investigation (He et al., 2023). Common plant species in sandy land areas include *Artemisia desertorum*, *Salix cheilophila*, *Corethrodendron fruticosum*, *Caragana korshinskii*, and *Amorpha fruticosa*. Additionally, mudflats and river valleys feature aquatic and halophytic vegetation including *Phragmites australis*, *Thermopsis lanceolata*, *Aster tataricus*, *Sueda salsa*, and *Neotrinia splendens*.

### 2.2 Experimental design and sample collection

According to different groundwater depth conditions (1–10 m), 20 sample sites were established to investigate the responses of plant diversity, soil properties, and bacterial communities to GTD changes in the Artem and Salix coverage areas. Groundwater table data were sourced from the Yulin Water Resources Bureau of Shaanxi Province. First, within each sample site, 5 m × 5 m quadrats were set up to assess plant diversity, including parameters such as plant coverage, plant height, and associated plant species. Subsequently, rhizosphere soil samples were obtained from a depth of 0–20 cm below the ground surface using a small sterile shovel. The ‘five point method’ was employed within the same quadrat, preceded by the removal of surface litter. Finally, three soil samples were collected from each quadrat after on-site mixing. All soil samples were collected from the rhizosphere. One section was allocated for the analysis of soil chemical and physical properties, while the remainder was preserved at −80°C to facilitate future soil DNA extraction. Soil sampling was conducted in July 2021, and subsequent testing was conducted within a two-week timeframe from the date of collection.

### 2.3 Measurement of soil properties

Soil water content (SWC, %) was measured using the drying method (constant temperature of 105°C, drying for 8 h). The soil pH was assessed using a soil extract with a soil–water ratio of 1:2.5. Soil organic carbon content (SOC, g/kg) was determined using the potassium dichromate oxidation external heating method (Nelson and Sommers, 1982). Total nitrogen (TN, g/kg) was quantified using the semi-micro-Kjeldahl method (Bremner and Mulvaney, 1982). Available phosphorus (AP, mg/kg) was determined using the NaHCO3 extraction-molybdenum antimony anti-colorimetric method (Olsen and Sommers, 1982).

### 2.4 High-throughput sequencing of microbial communities

Total soil DNA was extracted from the soil samples (0.5 g) using the OMEGA Soil DNA Kit (M5635-02) (Omega Bio-Tek, Norcross, GA, USA). The extracted DNA was qualitatively assessed via agar gel electrophoresis and its concentration and purity were determined using a NanoDrop NC2000 spectrophotometer (Thermo Fisher Scientific, Waltham, MA, USA). Subsequently, it was stored at −20°C to prevent evaporation. Amplification of the 16S rRNA gene was performed using primers 338F (5′-GCCAGCMGCCGCGGTAA-3′) and 806R (5′-CCGGACTA CHVGGGTWTCTAAT-3′), following the approach outlined. The PCR conditions consisted of 25 cycles, including pre-denaturation at 98°C for 2 min, denaturation at 98°C for 30 s, annealing at 55°C for 30 s, and elongation at 72°C for 5 min. After purification of the PCR products by gel electrophoresis, dsDNA was accurately quantified using Quant-iT PicoGreen dsDNA Assay Kit (Invitrogen, Carlsbad, CA, USA). The samples were homogenized based on the quantified values. The integrity of the DNA fragments was assessed using Illumina measurements from Shanghai Paisenor Biotechnology Co. LTD (Shanghai, China). The resulting DNA stock solution was stored at −20°C and diluted tenfold with ddH_2_O before being stored at 4°C. Each sample was analyzed in four replicates.

### 2.5 Data analysis

Plant community diversity and evenness indices were obtained from a study by Christian et al. (2022). Variations in the relative abundance of rhizosphere bacteria, alpha diversity, plant diversity, and edaphic variables in SW and DW were assessed using one-way analysis of variance (ANOVA). To investigate the impact of GTD on the composition of rhizospheric bacterial taxa, both alpha and beta diversities were calculated. Four specific indices were employed to comprehensively evaluate the alpha diversity of bacterial communities. Chao1 (Chao, 1984) represented richness, whereas Margalef, Shannon and Simpson indices denoted diversity. Pielou evenness index (Pielou, 1966) was used to characterize the evenness. Beta diversity was determined through Principal Coordinate Analysis (PCoA) based on unweighted UniFrac dissimilarity matrices, exploring differences in soil bacterial community composition between the two sandy plant species under SW and DW conditions.

The network structure was defined using igraph packages incorporating different topological parameters, such as modularity, average degree, and inter-community interactions. For the statistical analysis of rhizosphere soil bacterial community networks in both sandy plants across various GTD, igraph packages were used. Gephi software was used to visualize the co-occurrence networks (Bastian et al., 2009). Rhizosphere bacterial abundance, diversity, and functional bacteria were assessed under diverse GTD conditions through high-throughput 16S rRNA gene sequencing using the Functional Annotation of Prokaryotic Taxa (FAPROTAX) approach. Mantel tests were used to detect the connections between plant diversity, soil edaphic variables, microbial community structure, and functional microorganisms, utilizing the Personalbio Gene Cloud Platform.^1^

## 3 Results

### 3.1 Characteristics of plant communities and edaphic factors with GTD

**Figure 2:**
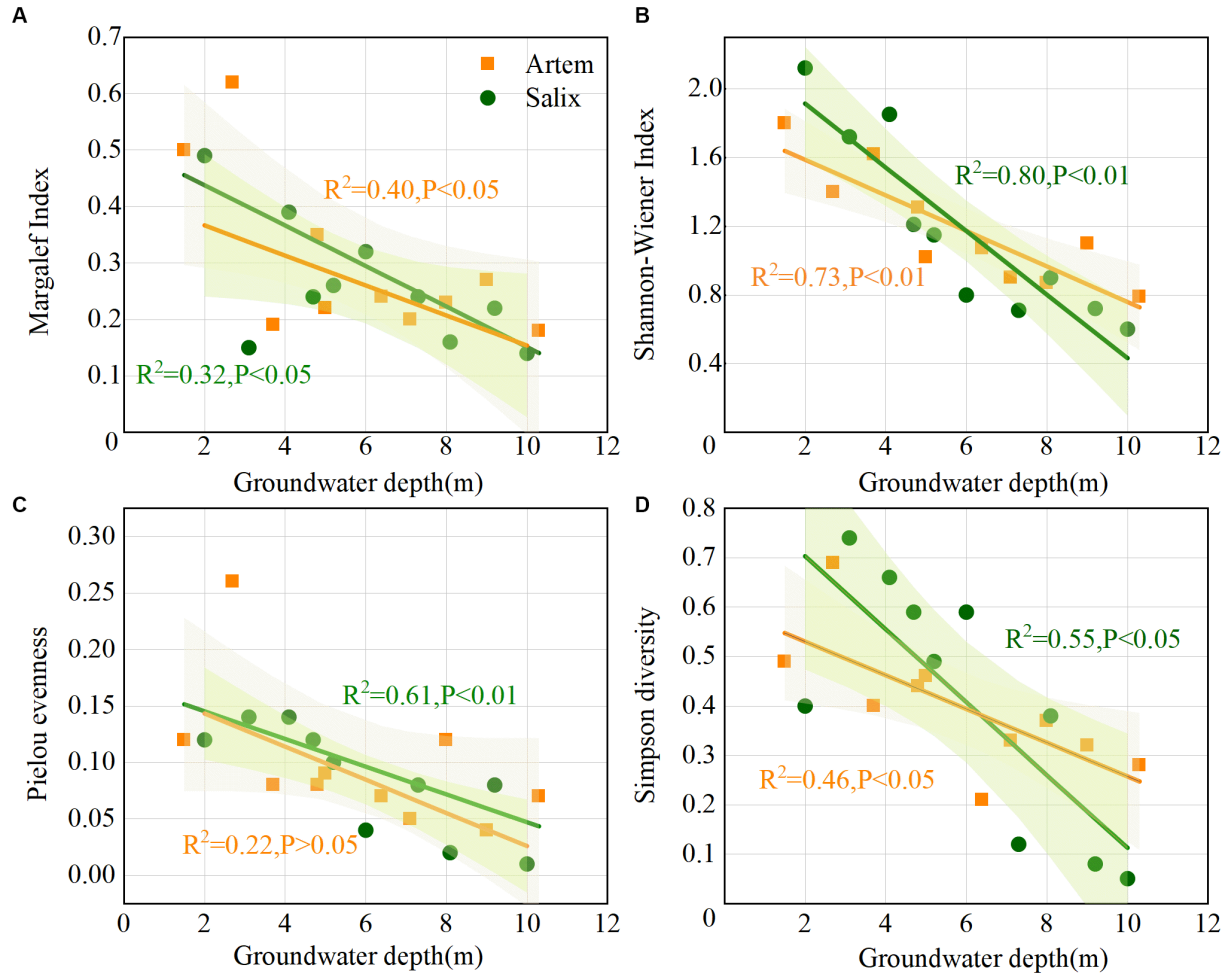
Variation of plant community with increasing groundwater table depth. Panels **(A-D)** represent Margalef, Shannon-Wiener, Pielou evenness, and Simpson diversity index of *Artem* and *Salix* sites, respectively.

All four diversity indices for both Artem and Salix displayed a decreasing trend as GTD increased (Figure 2). The Pielou index, reflecting evenness in species distribution, exhibited relatively low values, ranging from 0.04 to 0.26 (Artem) and 0.01 to 0.14 (Salix), indicating that the overall distribution of plant species in the sandy soil was not uniform (Figure 2C). The Margalef index, indicative of species richness, ranged from 0.18 to 0.62 (Artem) and 0.14 to 0.49 (Salix), indicating non-uniform species distribution in both Artem and Salix plots, with varying degrees of alteration (Figure 2A). Simpson’s index ranged from 0.21 to 0.69 (Artem) and 0.05 to 0.74 (Salix), indicating a relatively simplified community or habitat species structure and composition, coupled with high interspecific heterogeneity (Figure 2D). Moreover, the Shannon-Wiener index, which provides a comprehensive measure of species richness and homogeneity, exhibited a broad range, varying from 0.79 to 1.62 (Artem) and 0.6 to 2.12 (Salix) (Figure 2B). This wide variability demonstrates the significant impact of groundwater depth on plant growth in the Mu Us sandy soil.

**Figure 3:**
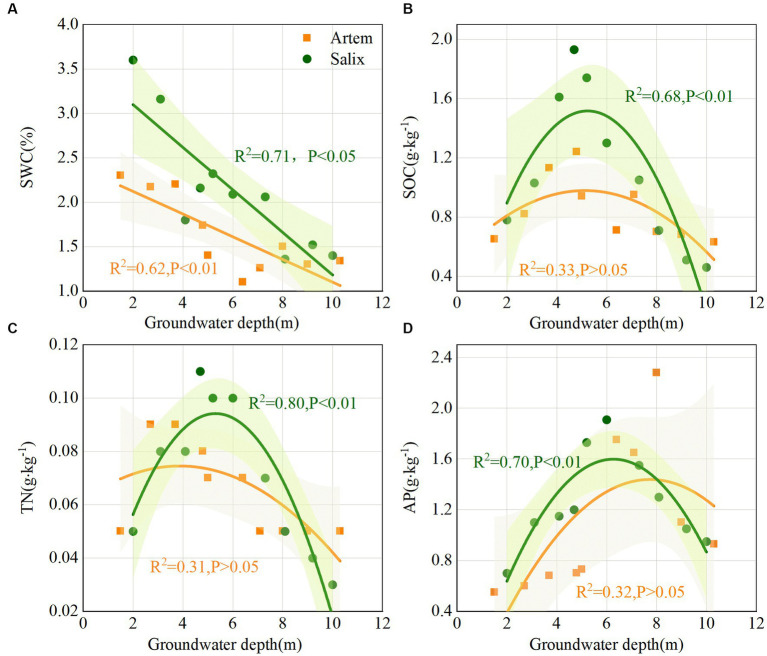
Changes in soil physicochemical properties of both plants with increasing groundwater table depth. Panels **(A-D)** represent soil water content (SWC), soil organic carbon (SOC), total nitrogen (TN), and Available phosphorus (AP) of *Artem* and *Salix* sites, respectively.

With increasing GTD, the SWC exhibited a downward trend at both plant sites. Notably, the SWC of Salix consistently exceeded that of Artem, and displayed an evident declining pattern (Figure 3A). This suggests that the rhizosphere soil of Salix has a higher water availability than that of Artem. Changes in soil nutrients (SOC, TN, and AP) and pH in response to GTD followed quadratic polynomial trends, indicating initial increments followed by subsequent declines. The nutrient content of Salix consistently surpassed that of Artem, suggesting a higher soil nutrient content of Salix (Figures 3B–D). In addition, the soil pH of both plants exhibited significant alterations within the GTD range of 4–6 m (Supplementary Figure S1), indicating the strong influence of GTD within this interval.

### 3.2 Characteristics of soil bacterial communities with GTD

Characteristic of bacterial community composition.

The soil bacterial communities exhibited significant differences between the sand-fixing plants under various GTD. Specifically, Artem and Salix ASVs were notably more abundant at a GTD < 5 m than GTD > 5 m (Supplementary Figure S2). Hence, it is verified that the groundwater depth of 5 m can be used as the dividing line between SW and DW. Although the abundance of Artem’s rhizospheric bacteria at the order, family, and genus levels were higher at GTD < 5 m than GTD > 5 m, the difference was not statistically significant. In contrast, the rhizosphere bacterial community of Salix at GTD < 5 m exhibited significantly greater species diversity at the phylum, class, order, and family levels (*p* < 0.05) than at GTD > 5 m (Supplementary Table S1). In summary, GTD of 5 m influenced both the quantity and species composition of rhizospheric microbial communities for both plants.

**Figure 4:**
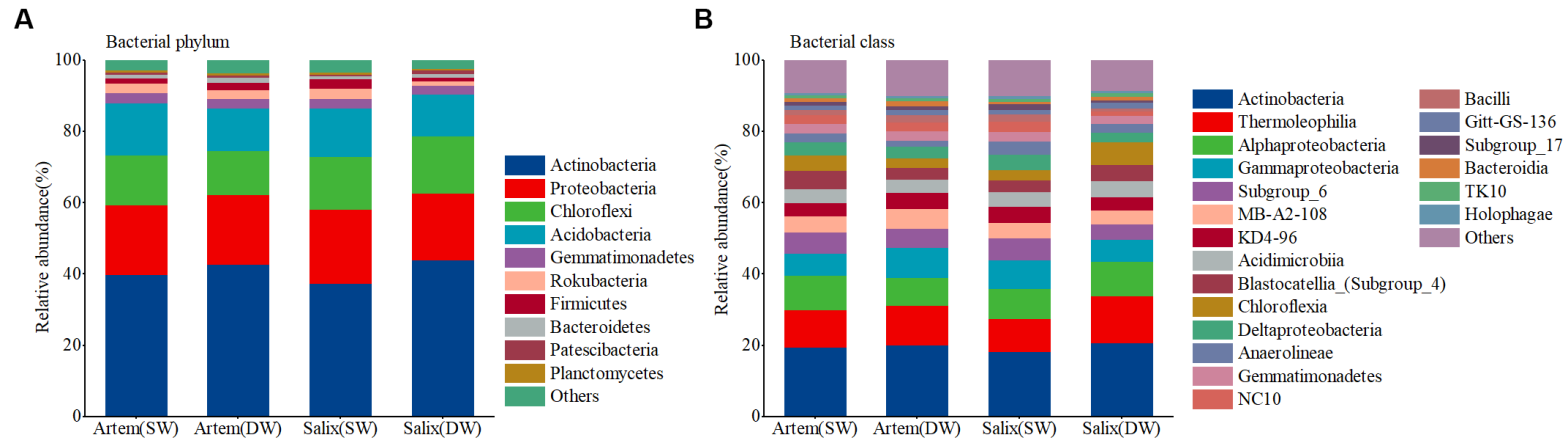
Relative abundance of soil bacterial communities at phylum **(A)** and class **(B)** levels.

The relative abundance of major bacterial phyla did not differ significantly between the rhizosphere bacterial communities of Artem and Salix under varying GTDs (Figure 4). The dominant phyla in the rhizosphere microbial community (with a relative abundance >1%) were Actinobacteria, Proteobacteria, Chloroflexi, and Acidobacteria, collectively constituting 86.49–90.47% of the total relative abundance. Specifically, the relative abundance of Chloroflexi in the rhizosphere bacterial community was significantly higher at DW Salix sites than at Artem sites, whereas the relative abundance of Acidobacteria was notably higher at SW Artem sites than at Salix sites (*p* < 0.05). Actinobacteria, Thermoleophilia, Alphaproteobacteria, and Gammaproteobacteria were the dominant bacterial classes (with a relative abundance >1%), collectively representing 44.06–49.72% of the total relative abundance (Supplementary Table S2). Significantly divergent trends were also observed in some major bacterial classes within the rhizosphere communities of Artem and Salix under different GTD, including Subgroup_6 (*p* < 0.05), Chloroflexia (*p* < 0.05), Deltaproteobacteria (*p* < 0.05), Anaerolineae (*p* < 0.05) and NC10 (*p* < 0.05) (Supplementary Table S3). These findings indicate that variations at the major bacterial classes were more pronounced than those at the phylum level in the rhizosphere soil of both plants in response to changing GTD. Consequently, the relative abundance of sandy soil bacteria was influenced by both the GTD and plant species.

#### 3.2.1 Changes in bacterial community diversity

**Figure 5:**
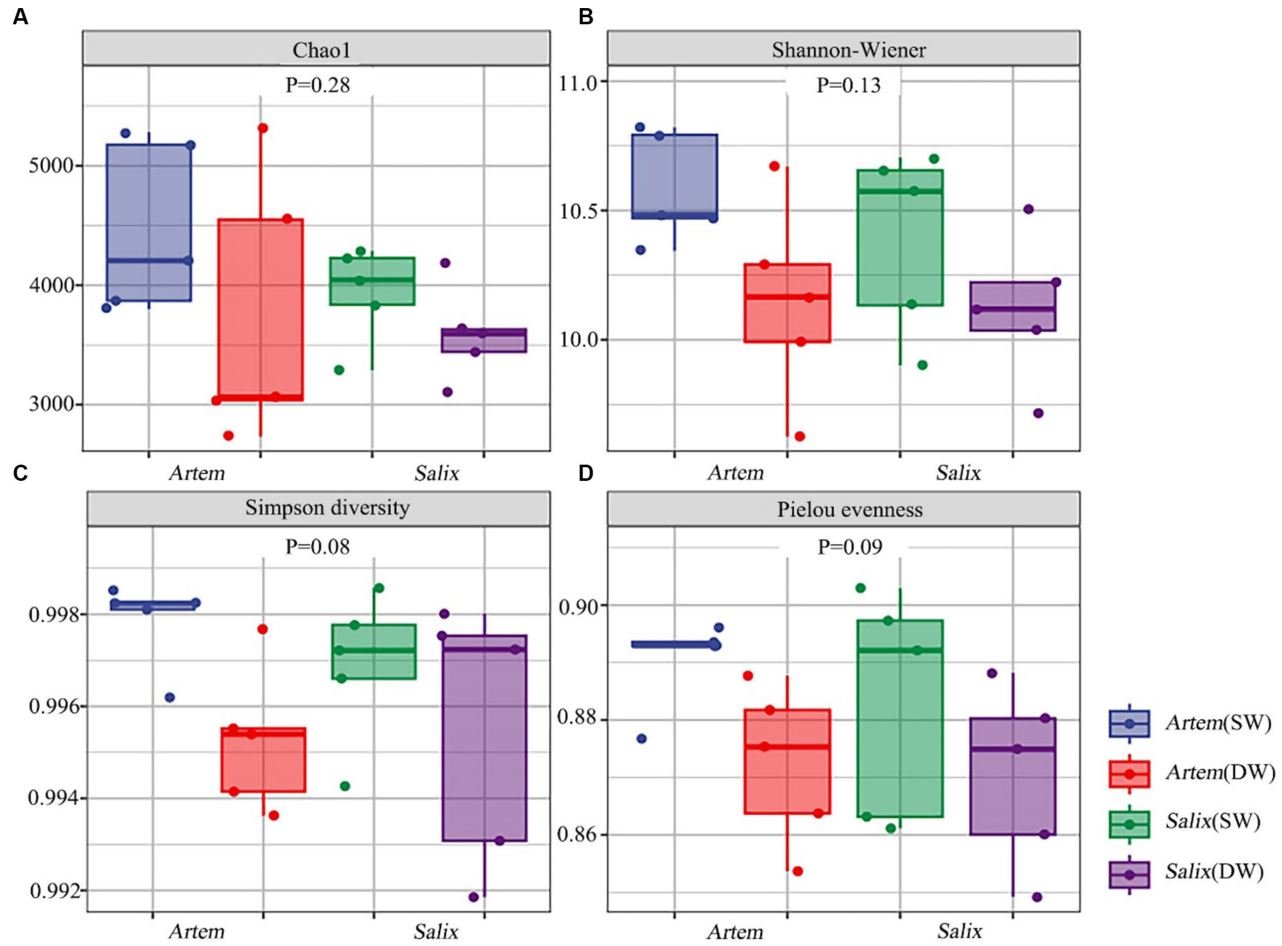
Alpha diversity of soil bacterial communities of two plants across different groundwater areas. Panels **(A-D)** represent Chao1, Shannon-Wiener, Simpson diversity and Pielou evenness index of rhizosphere bacteria community in the shallow groundwater table depth range (SW) and deep groundwater table range (DW) of Artem and Salix sites, respectively.

**Figure 6:**
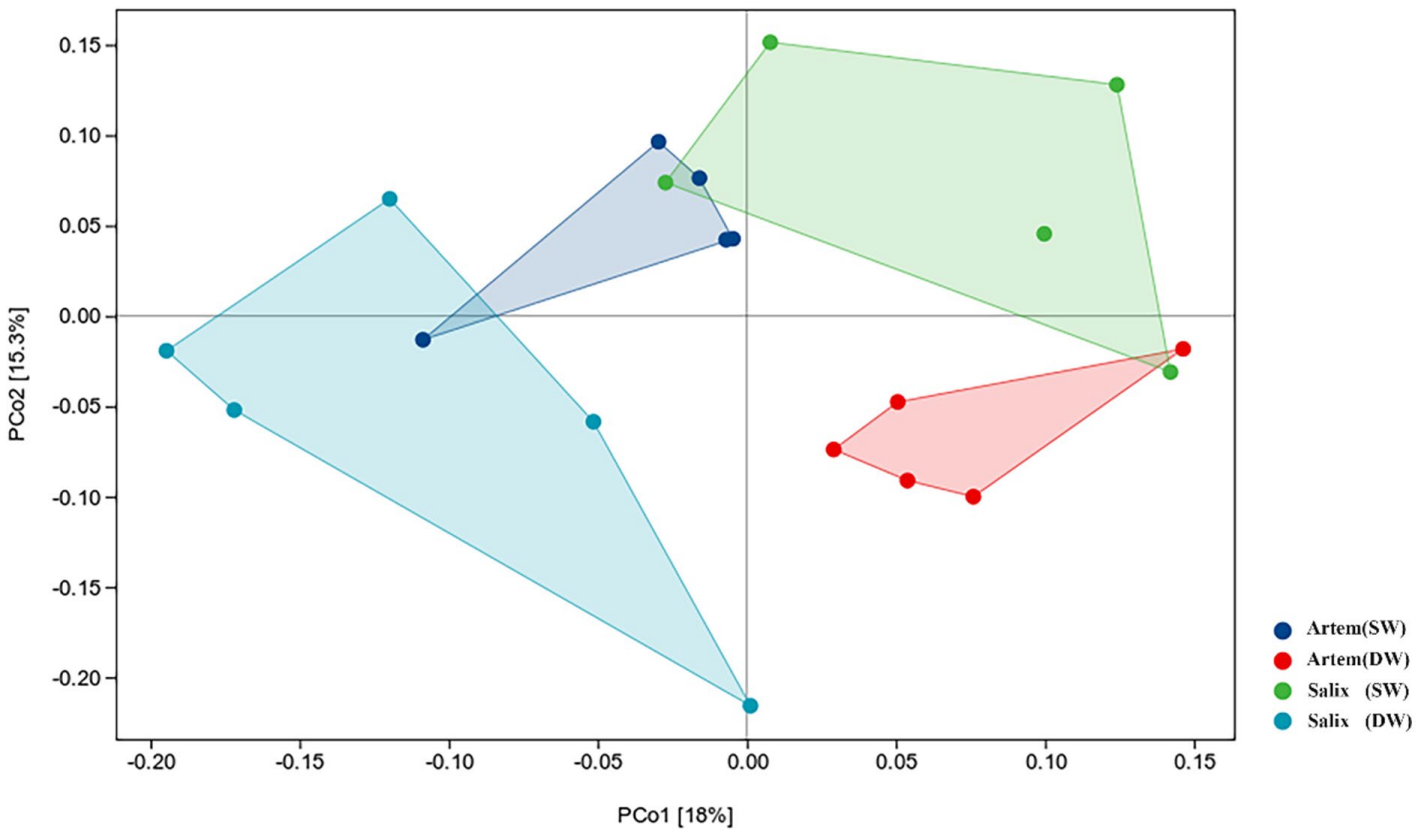
Ordination plot derived from principle coordinate analyses of weighted UniFrac distances of soil rhizosphere bacterial communities of two plants in varying groundwater conditions.

The alpha diversity (Chao1, Shannon-Wiener, Simpson diversity, and Pielou evenness index) of rhizosphere bacteria in both *Artem* and *Salix* were notably higher at GTD < 5 m (Figure 5). However, no significant differences in bacterial alpha diversity were observed between the SW and DW groups. The beta diversity of the soil bacterial community was further analyzed through principal coordinate analysis (PCoA) (Figure 6), complemented by statistical assessment via multiple analysis of variance (PERMANOVA) (Table 1). The first and second axes of the principal coordinate analysis explained 18 and 15.3% of the unconstrained ranking variance for rhizosphere bacterial communities across different GTD conditions in both Artem and Salix, respectively. Figure 6; Table 1 illustrate that using GTD = 5 m as the dividing line, the rhizosphere bacteria of Artem and Salix formed distinct and stable clusters in the SW and DW regions, respectively. Notably, the bacterial communities in the rhizosphere of the Artem (DW) sites were significantly different from those in the other groups (PERMANOVA: *F* = 2.00, *p* = 0.001).

**Table 1:** Differences in soil bacterial communities between the two plants across different groundwater areas (PERMANOVA).

Group 1	Group 2	Sample-size	Permutations	pseudo-F	Value of *p*	*q*-value
*Artem* (SW)	*Artem* (DW)	10	999	2.408023	0.012	0.024
*Artem* (SW)	*Salix* (SW)	10	999	1.275553	0.111	0.111
*Artem* (SW)	*Salix* (DW)	10	999	1.532867	0.022	0.033
*Artem* (DW)	*Salix* (SW)	10	999	1.850057	0.048	0.0576
*Artem* (DW)	*Salix* (DW)	10	999	2.378745	0.01	0.024
*Salix* (SW)	*Salix* (DW)	10	999	2.472371	0.01	0.024

“Permutation” represents the number of permutation tests, “pseudo-F” represents the value of the *F* statistic, “value of *p*” represents the value of *p* of the permutation test, and “*Q*-value” represents the q-value corrected for multiple tests.

#### 3.2.2 Changes in bacterial community structure

**Figure 7:**
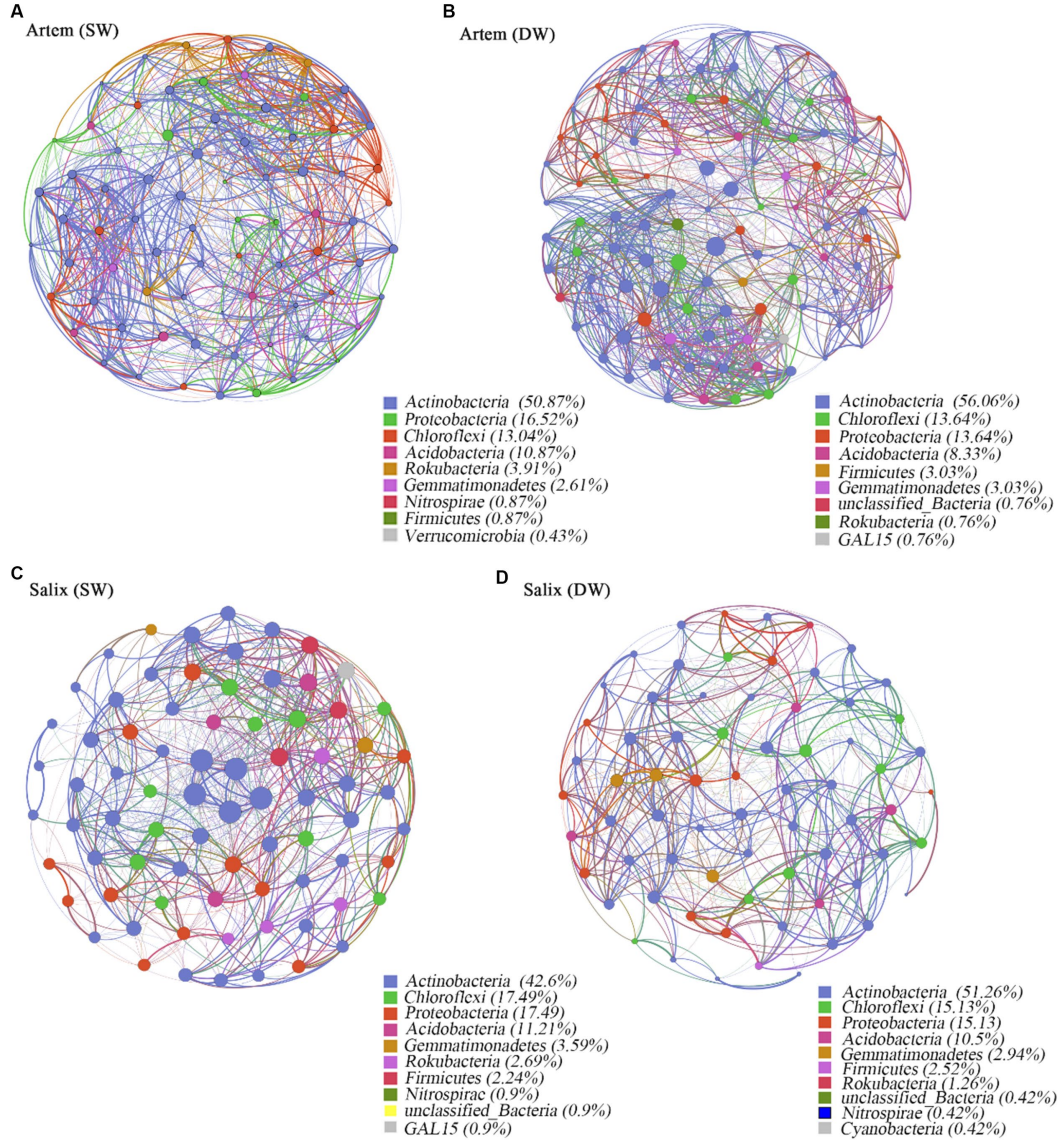
Panels **(A-D)** exhibit co-occurrence networks of soil rhizosphere bacteria community in the shallow groundwater table depth range (SW) and deep groundwater table range (DW) of Artem and Salix sites, respectively. Connections indicate significant correlations (Screening conditions: Relative abundance of bacterial phyla>0.004). The nodes are colored by phylum and represent an operational taxonomic unit (97% sequence identify threshold, ASV). The size of each node is proportional to the number of connections (degrees). The thickness of each connection between two nodes (edge) is proportional to the values of Spearman’s correlation coefficient.

Co-occurrence network analysis was used to investigate the ecological interactions among the microbial species in diverse environmental contexts. In this study, we constructed a co-occurrence network based on the microbial taxa at the ASV level of two plants subjected to varying groundwater depths (Figure 7). The results showed that the soil bacterial community of Artem in SW exhibited a more extensive and stable network structure, whereas the bacterial network of Salix in both SW and DW displayed a similar structure and was largely unaffected by changes in GTD. In the co-occurrence network, Actinobacteria, Proteobacteria, Chloroflexi, and Acidobacteria exhibited robust connectivity with the surrounding nodes, constituting 88.79–92.02% of all nodes (Figure 7).

The topological properties of the bacterial co-occurrence network are presented in Supplementary Table S4, which displays notable discrepancies below the threshold of 0.7. Specifically, at GTD < 5 m, Artem exhibited a greater number of network nodes and edges (230 nodes and 9,326 edges) than GTD > 5 m (132 nodes and 1961 edges). In the case of Salix, the number of network nodes and edges at GTD < 5 m (223 nodes and 5,518 edges) was nearly equivalent to that at GTD > 5 m (238 nodes and 6,095 edges) (Supplementary Table S4). Positive interactions accounted for 52.27 to 55.6%, while negative interactions ranged from 44.38 to 47.73% across different GTD levels. This suggests that the rhizosphere bacterial communities of both sand plants adapted to the arid soil conditions in the Mu Us region by engaging in mutual facilitation, coordination, predation, and antagonism, thereby forming a tight network. In the *Artem* co-occurrence network at a GTD < 5 m, the total number of links, mean node degree, complexity index, and mean path length were higher, indicating shorter links between nodes, a higher degree of linkage, greater complexity, and a more stable rhizosphere bacterial community. However, *Salix* exhibited distinct topological characteristics. In both the SW and DW, the total number of connections, average node degree, average path length, and complexity index in the co-occurrence network were similar. This further underscores the robust adaptability of the rhizosphere microorganisms in Salix to fluctuating groundwater table.

Four nodes meeting the criteria (Zi ≥ 2.5, Pi ≤0.62) were selected within the module hubs through an assessment of intra-module and inter-module connectivity within the bacterial community co-occurrence network. These nodes, specifically ASV17731, ASV187664, and ASV233705, were identified within the Artem rhizosphere bacterial co-occurrence network in the DW (Supplementary Figure S3). Additionally, ASV192677 was identified in the Salix rhizosphere bacterial co-occurrence network in the SW. Notably, these four keynote species were from *Actinobacteria, Firmicutes, and Acidobacteria*. ASV17731 and ASV192677 belong to the Subgroup_17 class of *Actinobacteria*, representing significant species in this phylum. They are associated with soil fertility enhancement due to their capacity for organic matter decomposition. ASV187664, a member of the phylum *Firmicutes*, participates in soil respiration metabolism and chemoheterotrophic processes, demonstrating resilience under harsh environmental conditions. ASV233705, which originates from *Catenulispora* within *Actinobacteria*, serves as a nutrient provider to plant roots, ensuring optimal plant growth.

#### 3.2.3 Changes in functional bacterial communities

**Figure 8:**
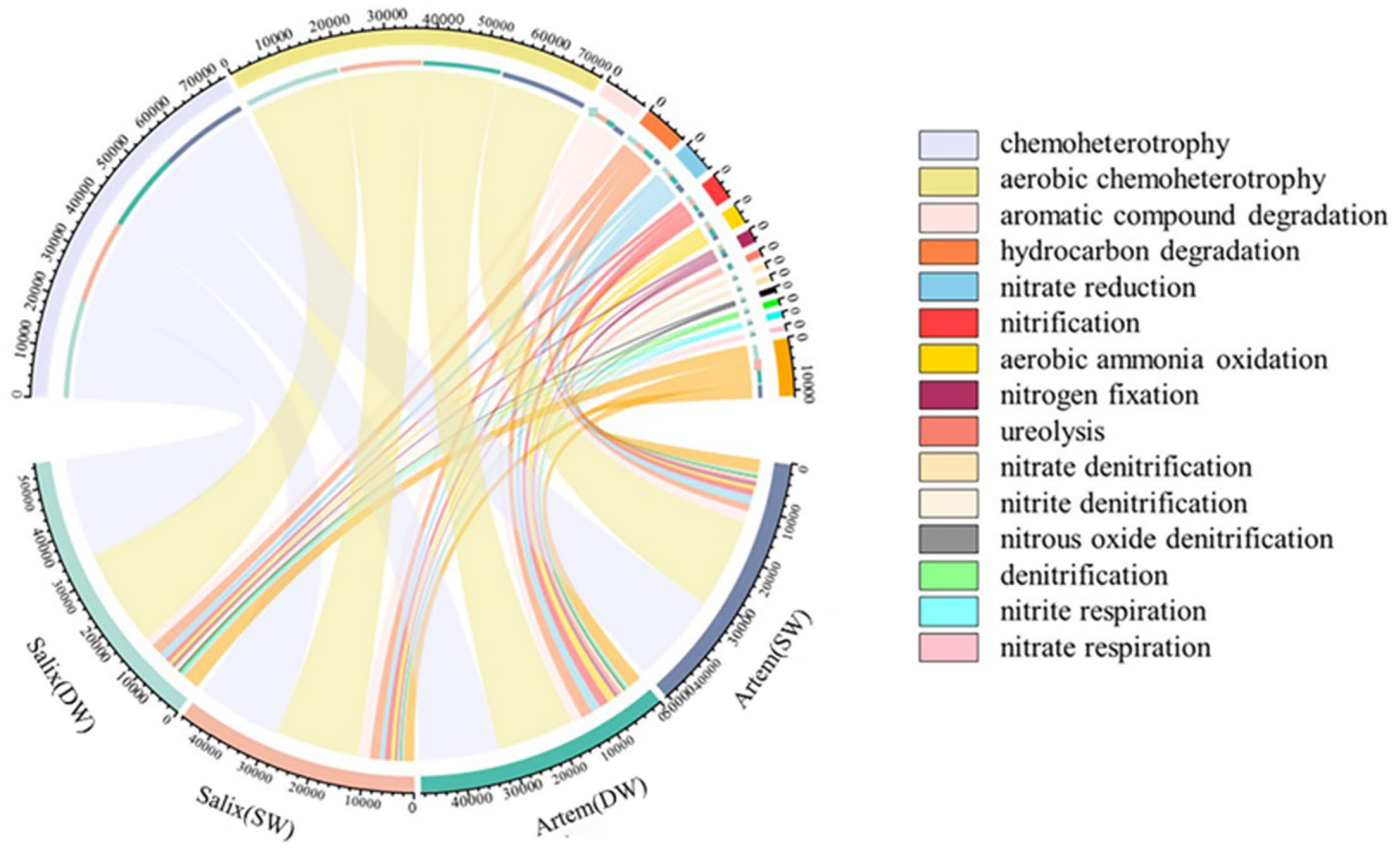
Prediction and analysis of soil bacterial community functions at various groundwater depths.

A chord diagram depicting the relative abundances of the top 15 functional bacteria was generated (Figure 8). The functions exhibited by the rhizosphere bacterial communities of Artem and Salix under varying GTD primarily encompassed chemoheterotrophy (33.55–37.77%), aerobic chemoheterotrophy (33.12–37.24%), aromatic compound degradation (3.15–5.31%), hydrocarbon degradation (2.84–4.93%), nitrate reduction (2.22–3.95%), nitrification (1.74–3.92%), aerobic ammonia oxidation (1.37–3.39%), and nitrogen fixation (1.06–1.79%). Eight functional microbial categories with relative abundance exceeding 1% were identified.

**Figure 9:**
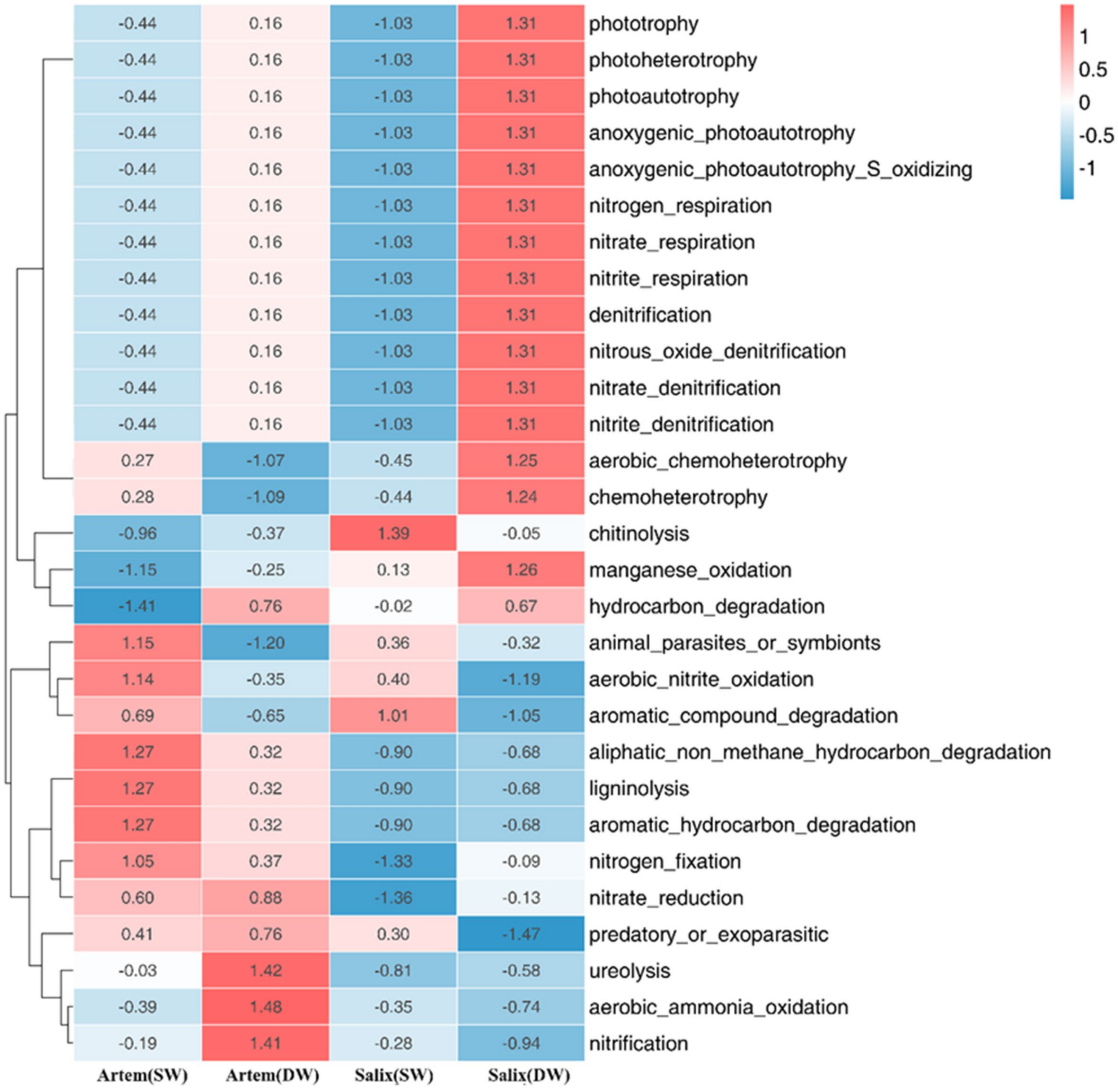
Soil functional bacteria of two plants across different groundwater depths.

Subsequently, the functional characteristics of the rhizosphere bacterial communities in both plant species exhibited significant variations in response to GTD (Figure 9). In the rhizospheric soil of Artem, there was a notable increase in the relative abundance of bacteria engaged in carbon metabolism, aliphatic non-methane hydrocarbon degradation, ligninolysis, aromatic hydrocarbon degradation, and other functional categories under SW conditions. Conversely, functional bacteria associated with the nitrogen cycle, including ureolysis, aerobic ammonia oxidation, and nitrification, showed a marked elevation in their relative abundance under DW conditions.

In the Salix rhizospheric soil, there was a noteworthy increase in the relative abundance of functional bacteria involved in the carbon cycle, including chitinolysis and aromatic compound degradation, under SW conditions. Conversely, under DW conditions, a significant increase in relative abundance was observed for functional bacteria associated with phototrophy and nitrogen metabolism, including phototrophy, photoheterotrophy, photoautotrophy, anoxygenic photoautotrophy, aerobic chemoheterotrophy, chemoheterotrophy, nitrogen respiration, nitrate respiration, nitrite respiration, denitrification, nitrous oxide respiration, nitrate denitrification and nitrite denitrification.

The results revealed that both *Artem* and *Salix* exhibited a higher presence of functional bacteria related to carbon metabolism in SW. However, in DW, *Artem* demonstrated a greater abundance of functional microorganisms linked to nitrogen cycle, whereas *Salix* displayed a higher proportion of functional microorganisms involved in the nitrogen cycle and phototrophic processes.

### 3.3 Response of rhizosphere bacterial communities to plant–soil factors across various GTD

**Figure 10:**
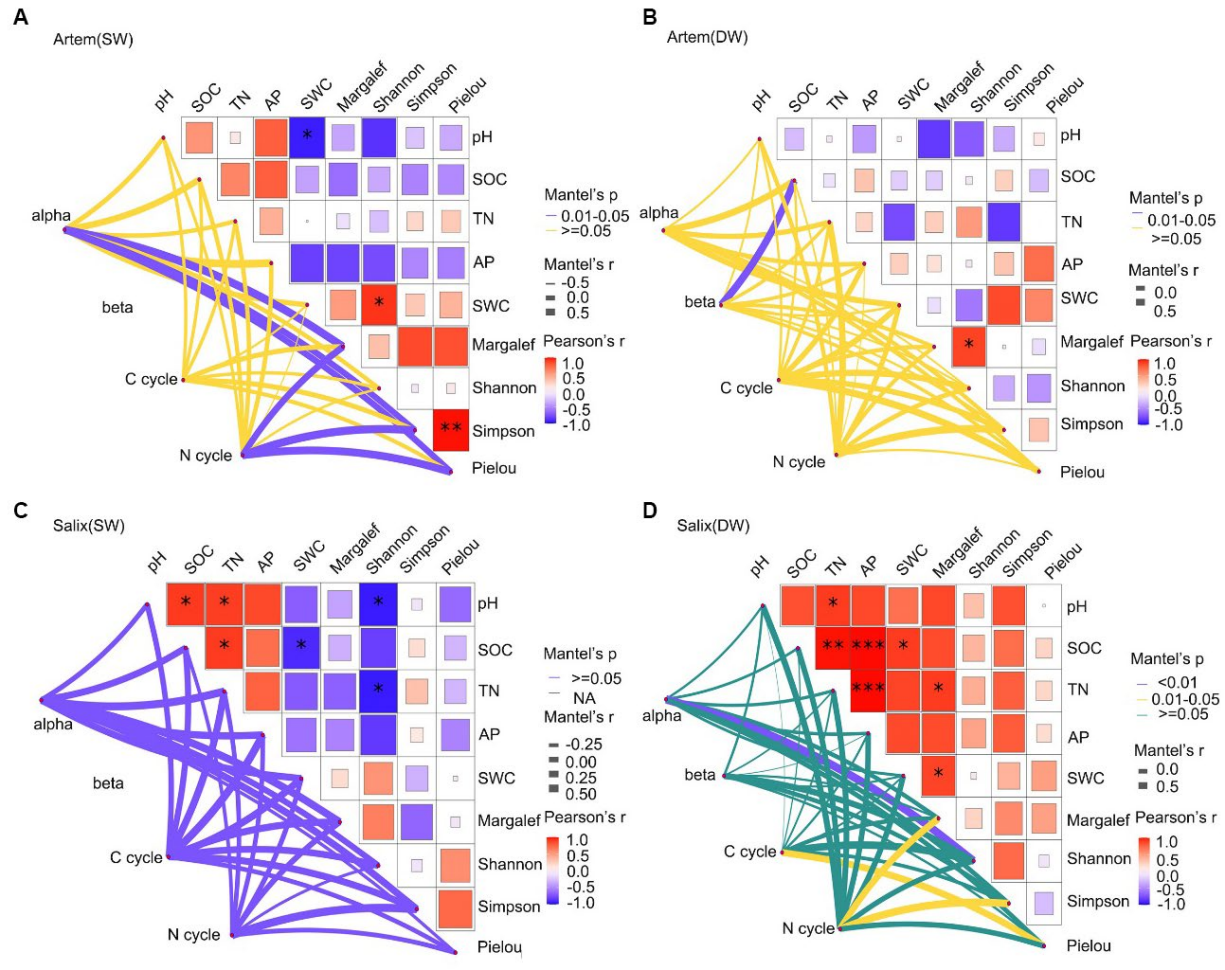
**(A-D)** show the shallow relationship between plant, soil factors and major functional microbes in the shallow groundwater table depth range (SW) and deep groundwater table range (DW) of *Artem* and *Salix* sites, respectively.

The Mantel test correlation analysis (Figure 10) revealed a significant positive correlation between plant and soil factors and the microbial community. Specifically, the SWC exhibited a remarkable association with the Shannon-Wiener diversity index of the Artem, which may exerted a prominent influence on bacterial alpha diversity (Figure 10A). This indicated that plant growth in SW was highly dependent on SWC. Moreover, enhanced plant diversity promoted greater involvement of functional microorganisms in facilitating plant nutrient uptake, thereby leading to an increase in alpha diversity within the rhizosphere bacterial community. Afterwards, plant diversity indices (Margalef, Simpson Wiener diversity, and Pielou evenness) demonstrated a substantial positive impact on functional bacteria that participate in the soil nitrogen cycle. Hence, SWC affected both rhizosphere bacterial diversity and the presence of functional microorganisms integral to the nitrogen cycle, all through its effects on plant growth under SW conditions. In contrast, in DW, except for the notable effect of SOC on microbial beta diversity, there was no correlation between plant and soil factors. Furthermore, these factors did not significantly affect the functional microorganisms involved in carbon and nitrogen cycles (Figure 10B). This implies that SOC exerts a more pronounced effect on the rhizospheric bacterial diversity of the Artem in DW conditions. And this observation result aligns with the identification of critical species, particularly the representative *Actinobacteria*, specifically ASV17731, from the Subgroup_17 class, known for their association with soil fertility and their role as decomposers of soil organic matter.

In Salix sites subjected to SW conditions, although plant and edaphic factors did not significantly affect microbial diversity and functional composition, a noteworthy correlation was detected between almost all soil–plant factors (except the Pielou index) and functional microorganisms engaged in the C cycle compared to the N cycle (Figure 10C). As far as Salix sites under DW conditions was concerned, not only plant diversity (Shannon diversity, Pielou evenness index) was strongly correlated with the C cycle functional microorganisms, but both plant diversity (Margalef, Simpson diversity index) and soil nutrients (pH, SOC, TN, AP and SWC) also had a significant effect on the N cycle (Figure 10D). These findings were similar with earlier microbial function predictions, affirming that rhizosphere microbes in Salix played a facilitative role in the carbon cycle under SW conditions. This could be attributed to representative species of Actinomycetes, specifically ASV192677 from the Subgroup_17 order, which contributed to soil fertility enhancement and acted as decomposers of soil organic matter. This, in turn, contributes to soil carbon cycling. Notably, these microbes made substantial contributions to both carbon and nitrogen cycling under DW conditions.

## 4 Discussion

### 4.1 Responses of plant diversity and soil physicochemical factors to GTD

The microtopographic features of the aquifer structure in Mu Us Sandy Land contributed to the variation in the GTD. As the GTD increased, the overall diversity of plant communities exhibited a decrease trend (Figure 2). This aligns with the study by Chen et al. (2006) in the Tarim River Basin, where increasing GTD led to a decrease in species diversity and richness, along with an overall low Pielou evenness index. In the study area, dominant vegetation primarily comprised shrubs, including Salix cheilophila, Artemisia desertorum, and Corethrodendron fruticosum, along with herbs, such as *Phragmites australis*, Thermopsis lanceolata, and *Aster tataricus* in the SW. In contrast, the DW area was characterized by shrubs such as Salix and Artem, accompanied by species such as *Lespedeza bicolor* and saplings of *Pinus sylvestris* var. mongolica. This pattern indicated fewer plant species and a simpler community structure in DW, reflecting soil water stress as the principal driver of changes in vegetation species diversity. This was attributed to the linear relationship between the GTD and SWC (Figure 3A), which is consistent with the findings of Zhang et al. (2018). In general, when the GTD is shallower, the root zone benefits from recharging via rising capillary water, thereby maintaining a higher soil water content. Conversely, with a deeper GTD, the soil receives less recharge from rising capillary water, resulting in lower soil water content. This indicates that groundwater and soil moisture dynamics are pivotal factors that affect vegetation growth (Huang et al., 2019; Barron-Gafford et al., 2021; Ma et al., 2022).

Previous studies have demonstrated that water efficiency in arid regions has notable implications for soil nutrient sustainability (Zeng J. et al., 2020; Condon et al., 2021; Reed et al., 2022). In both Artem and Salix sites, soil nutrients increased first and then decreased with the increase of GTD (Figures 3B–D). On the one hand, when the groundwater table is shallow, higher soil moisture levels reduce oxygen penetration into the soil. This reduces the activity of aerobic microorganisms involved in decomposition, resulting in a lower rate of organic matter breakdown and consequent soil nutrient accumulation (Sahrawat, 2003). On the other hand, under SW conditions, an adequate supply of vegetative nutrients is facilitated by hydraulic uplift within the root system (Prieto et al., 2012). This may explain why soil nutrient levels increased as GTD increased in SW. Conversely, as the groundwater table continued to decrease, reduced soil water content and heightened aeration in the vadose zone accelerated the decomposition of soil organic matter, leading to a decrease in SOC. This aligns with the trend reported by Zhao et al. (2023) for the Horqin sand plain, where nutrients exhibited a decline with increasing GTD. In this study, SOC, TN, and AP exhibited an initial increase, followed by a decrease in the GTD (Figure 3C). There are two potential explanations. First, TN and AP contents followed a similar trend to SOC with decreasing GTD, likely because 95% of TN and 40 to 60% of AP were derived from organic matter, thereby linking the decrease in SOC content to TN and AP levels (Wu et al., 2018). Second, this phenomenon may also be associated with the trend in vegetation diversity, which demonstrated a significant decrease trend (Figure 2), indirectly affecting the nutrient change. This implies that alterations in environmental factors, particularly groundwater table depth, in drylands exert varying influence on functional traits at the plant community level, potentially modifying their nutrient utilization and storage strategies (Reich, 2014). This also indicates that soil nutrient variations are contingent on GTD, plant cover, and their interactions (Li et al., 2021). In addition, SWC, SOC, TN, and AP levels in Salix surpassed those in Artem (Figure 3). Since Salix owns more extensive and robust root system, which confers greater efficiency in water and nutrient uptake than Artem (Chen et al., 2022). In arid sandy soils, shrubs typically exhibit well-developed root systems that mitigate water limitations (Xiao-Dong et al., 2022). This advantage facilitates nutrient enrichment around the plant root system through the ‘fertile island effect’, yielding pronounced spatial heterogeneity in soil physicochemical properties, particularly in nutrient content (Wang et al., 2019; Gao et al., 2022). Consequently, the groundwater table depth and vegetation patterns tend to influence nutrient accumulation in unsaturated soils (Zhang et al., 2022), thereby modifying the microenvironment, including factors such as pH, through alterations in soil redox processes and cation exchange rates (Supplementary Figure S1).

### 4.2 Response of rhizosphere bacterial community structure and co-occurrence networks to GTD

As depicted in Figure 4, the predominant phyla (with a relative abundance exceeding 1%) within the soil bacterial communities under varying GTDs for both plants included Actinobacteria, *Proteobacteria*, *Chloroflexi*, and Acidobacteria (Supplementary Table S2). The principal classes (relative abundance >1%) were Actinobacteria, Thermoleophilia, Alphaproteobacteria, and Gammaproteobacteria (Supplementary Table S3). Although the proportions of the major taxa varied, the major dominant taxa were basically the same as in other studies (Bi et al., 2021; Li et al., 2021). Similarly, the co-occurrence network within the rhizosphere bacterial communities of Artem and Salix revealed distinct compositions in the SW and DW. Specifically, in SW, the network was primarily composed of Proteobacteria and Acidobacteria, whereas Actinobacteria played a prominent role in DW (Figure 7). The observed impact of the GTD on the dominant bacterial phylum was notable. Specifically, alterations in the relative abundance of bacterial communities revealed a notable prevalence of the phylum Proteobacteria and its associated classes within the rhizosphere soil bacterial community (Figure 7). This phylum consists of four major groups (α-Proteobacteria, β-Proteobacteria, δ-Proteobacteria, and γ-Proteobacteria), all of which are Gram-negative, indicating their propensity to accumulate in the rhizosphere and facilitate organic matter decomposition. The presence of *Acidobacteria*, which produce extracellular polysaccharides that facilitate prokaryotic adhesion to root surfaces, demonstrates their positive interaction with plants, ultimately promoting plant growth (Kielak et al., 2016). In DW, the Artem co-occurrence network identified ASV187664, ASV17731, and ASV233705 from Bacillus of Firmicutes, Subgroup_17, and Catenulispora of Actinobacteria, respectively (Supplementary Figure S3). Gram-positive bacteria play pivotal roles in nutrient cycling processes, including organic matter decomposition and chemoenergetic heterotrophy. This suggests that the heightened GTD places Artem in an inhospitable environment, compelling key functional microorganisms involved in nutrient cycling to extract nutrients from soil. Zeng L. et al. (2020) corroborated that Actinobacteria and Acidobacteria, which function as saprophytic bacteria, actively participate in organic carbon degradation and contribute to carbon cycling, with Acidobacteria demonstrating a preference for promoting plant growth in nutrient-scarce soil environments (Fierer, 2007). In contrast, Salix exhibited selection for only one critical species from Actinobacteria, ASV192677, associated with soil fertility in SW (Figure 8), suggesting that Salix may display greater resilience to GTD variations than Artem in SW, consequently exerting a more pronounced influence on limiting GTD.

Under SW conditions, the relative abundance of *Deltaproteobacteria*, *Anaerolineae*, and *NC10* class exhibited significant elevation compared to DW, indicating that specific families within *Deltaproteobacteria*, such as *Comamonadaceae*, are adept at utilizing nitrate-derived nitrogen for denitrification, thereby contributing to nitrogen cycling in SW (Khan et al., 2002). Furthermore, the relative abundance of Chloroflexia in the rhizosphere bacteria of Artem was notably higher in SW than in DW, whereas in Salix, the trend was reversed. Both Anaerolineae and Chloroflexia belong to the phylum Chloroflexi, characterized as anaerobic organisms that do not engage in oxygenic photosynthesis or nitrogen fixation. Instead, they fix carbon dioxide through the 3-hydroxypropionate pathway and are well suited for higher temperatures (Sprague et al., 1981). Therefore, the prevalence of Chloroflexia in SW of Artem may be attributed to its adaptation to elevated temperatures, whereas in DW of Salix, it may contribute to carbon fixation.

In the SW of Artem, there is relatively high microbial diversity and activity owing to the ample availability of essential nutrients for plant growth. Consequently, the soil bacterial community exhibited a more intricate network structure (Figure 7; Supplementary Table S4) and displayed heightened resilience to environmental fluctuations (de Vries et al., 2018; Tao et al., 2018; Luo et al., 2023). Furthermore, microbial networks characterized by increased co-occurrence and complexity tend to positively affect microbial behavior in nutrient cycling and subsequent ecosystem functioning (Wagg et al., 2019). This is attributed to their greater efficiency in resource utilization and information exchange (Faust, 2021; Ma et al., 2021). In contrast, for Salix, regardless of whether it was in SW or DW conditions, the complexity of the co-occurrence network did not exhibit significant differences. This implies that rhizosphere microorganisms in Salix demonstrated higher adaptability to changes in water table levels than those in Artem. Rhizosphere effect refers to the phenomenon that the exudates of plant roots and tissues provide abundant nutrients and energy for rhizosphere microorganisms. Therefore, this adaptability was likely attributed to the well-developed root system of Salix, which amplified the ‘rhizosphere effect’ in regulating microbial structure and influencing nutrient cycling (López et al., 2023). Furthermore, the co-occurrence network displayed a prevalence of positive connections over negative ones, indicating that microbial cooperation outweighs competition in adapting to the challenging conditions of arid desert ecosystems.

### 4.3 Regulation of soil nutrients by microbial community changes in Mu Us Sandy Land

In the SW of *Artem* sites (Figure 10A), further analysis can be conducted on the effects of plant diversity and soil factors on the diversity of rhizosphere microorganisms, as well as functional microorganisms engaged in carbon and nitrogen cycling. This indicates a potentially higher nitrogen content in the rhizosphere soils of *Artem* under SW conditions. In contrast, in DW conditions, there was minimal correlation between plant and soil factors, with only organic carbon significantly affecting microbial beta diversity. The substantial increase in GTD significantly affects various soil properties, including pH, SWC, and oxygen availability, thereby exerting selective pressure on the microbial community (Baselga, 2010). Consequently, microbial-mediated partitioning of carbon and nitrogen in the rhizosphere community becomes susceptible to change through alterations in critical species and functions crucial for sustaining material and energy production under adverse conditions (Yerbury et al., 2005; Bhattarai et al., 2015; Ghezzehei et al., 2019). For example, in response to drought stress, bacteria undergo increased synthesis of amino compounds to maintain intracellular osmotic pressure and prevent dehydration, thereby consuming substantial amounts of C and N resources (Schimel et al., 2007). Notably, the rate of carbon decomposition tends to increase with shallower groundwater tables, leading to heightened CO2 fluxes (Burkett and Kusler, 2000). This could potentially shift the status of the sandy soil from a carbon sink to a carbon source. This hypothesis is supported by the key species identified within the co-occurrence network, particularly the representative *Actinomyces* species *ASV17731* and *ASV233705*. These species are associated with soil fertility and function as decomposers of soil organic matter. In addition, ASV187664 from *Bacillus* of phylum *Firmicutes* exhibits resilience in extreme environments and plays a role in respiratory metabolism and chemoenergetic heterotrophy.

In the case of *Salix*, nearly all plant–soil factors demonstrated robust correlations with functional bacteria involved in the C cycle at GTD < 5 m (Figure 10). This suggests that the rhizosphere soil of *Salix* in SW may offer a more conducive environment for the survival of functional microbes associated with the C cycle. Specifically, the representative species ASV192677 from the *Actinomycete Subgroup_17 class* played a role in enhancing soil fertility through its involvement in organic matter decomposition. This is in line with the findings of Bi et al. (2021) in the Yulin Botany Garden, where pH, a crucial regulatory factor governing microbial community distribution, significantly affected the dynamic processes of the soil bacterial community. Furthermore, SWC, pH, SOC, nutrients, and litter have been reported to be significantly correlated with microbial communities in the arid and semi-arid regions of northern China (Li X. Q. et al., 2019; Na et al., 2019; Yang et al., 2019). Notably, under DW conditions, plant diversity not only displayed a robust correlation with the C cycle, but both plant diversity and soil nutrients also exerted a notable effect on the N cycle. This was consistent with the results predicted by the microbial function (Figure 9), suggesting that GTD affected the dynamics of carbon and nitrogen cycling. Carbon nutrient availability may be related to functional microorganisms, whereas alterations in the nitrogen content may involve more intricate processes. Chen et al. (2012) observed that the lowering of the groundwater table induced vigorous nitrogen mineralization, leading to an accumulation of ammonium in the soil. This can provide more effective nitrogen for the soil and stimulate the participation of functional microorganisms in the nitrogen cycle. This further indicates an effect on nitrogen recovery in the root zone in the deeper groundwater tables. In addition, certain studies have indicated that limited oxygen supply in the soil can markedly affect gaseous nitrogen losses (specifically N_2_O and N_2_ emissions). Under highly anoxic conditions facilitated by a shallow groundwater table, there is a propensity for enhanced N2O consumption, consequently reducing the total N2O emissions (Hansen et al., 2014). Given that optimal conditions for complete denitrification of N2 are typically identified when the oxygen supply is restricted, the presence of Soil Water (providing an anaerobic environment) has been suggested to potentially diminish soil N2O emissions while augmenting N2 emissions (Flessa et al., 1998).

## 5 Conclusion

We conducted a study investigating the correlation between rhizosphere bacterial communities and soil nutrition in the Mu Us Sandy Land, focusing on two representative sandy plants subjected to varying GTD. Our findings revealed distinct characteristics between *Salix* and *Artem* sites. *Salix* sites exhibited higher SWC levels and soil nutrient concentrations than *Artem* sites, suggesting greater adaptability of *Salix* to fluctuations in groundwater table. Notably, the co-occurring network of functional bacteria in the rhizosphere of the *Artem* sites was more intricate in SW, displaying heightened resilience to the environment. This complexity exerts a substantial influence on regulating the microbial structure and affecting nutrient cycling. In contrast, *Salix* demonstrated a lower responsiveness to changes in GTD, attributed to its well-developed root system, consequently amplifying the ‘rhizosphere effect’ in governing microbial structure and influencing nutrient cycling. In terms of functional rhizosphere bacteria, both *Artem* and *Salix* positively affected carbon metabolism in SW. Conversely, in DW, the rhizosphere bacterial community of *Artem* was found to be more conducive to nitrogen cycling, whereas *Salix* exhibited a dual promotion of both nitrogen cycling and phototrophic functions. Importantly, we found that the rhizosphere soil of SW had a higher carbon content at *Artem* sites, as GTD increases, the rate of organic carbon decomposition accelerates, potentially causing a shift from carbon sink to carbon source. *Salix* sites in SW contain higher carbon levels and consumption of greenhouse gas N_2_O, as GTD increases, the soil undergoes significant nitrogen mineralization, increasing soil nitrogen effectiveness. These findings indicate the crucial role of microorganisms in mediating carbon and nitrogen redistribution in the soil to mitigate the adverse conditions caused by fluctuating groundwater table depths. It is imperative to acknowledge that groundwater tables have a notable impact on greenhouse gas emissions from sandy rhizosphere soils. Further research is warranted to broaden our understanding of the various vegetation types.

## Data availability statement

The raw data supporting the conclusions of this article will be made available by the authors, without undue reservation.

## Author contributions

LH: Data curation, Formal analysis, Methodology, Visualization, Writing – original draft. XL: Funding acquisition, Project administration, Supervision, Writing – review & editing. RJ: Data curation, Methodology, Visualization, Writing – review & editing. YM: Data curation, Funding acquisition, Investigation, Writing – review & editing. PW: Investigation, Resources, Writing – review & editing. QC: Investigation, Resources, Writing – review & editing. YX: Investigation, Resources, Writing – review & editing.

## Footnotes

^1^https://www.genescloud.cn/home
