# Supplementary material for: Indirect regulation of topsoil nutrient cycling by groundwater depth: impacts on sand-fixing vegetation and rhizosphere bacterial communities
Source: Front Microbiol. 2023 Dec 5;14:1285922. doi: 10.3389/fmicb.2023.1285922 (PMC10746847; doi:10.3389/fmicb.2023.1285922)
Supplement: Supplementary file 1 [file Data_Sheet_1.docx]

Supplementary Material

Groundwater depth regulates soil nutrient cycling indirectly by affecting vegetation and rhizosphere microorganisms

**Lianyi Hao^a,b,c^, Xiuhua Liu^a,b,c^, ruiqing Ji^a,b,c^, Yandong Ma^d^, Puxia Wu^d^, Qingxi Cao ^d^, Yunling Xin^d^**

^a^ Key Laboratory of Subsurface Hydrology and Ecological Effect in Arid Region of Ministry of Education, Chang’an University, Xi’an 710054, China;

^b^ School of Water and Environment, Chang’an University, Xi’an 710054, China;

^c^ Key Laboratory of Eco-hydrology and Water Security in Arid and Semi-arid Region of Ministry of Water Resources, Chang’an University, Xi’an 710054, China;

^d^ Key Laboratory of State Forest Administration on Soil and Water Conservation & Ecological Restoration of Loess Plateau, Shaanxi Academy of Forestry, Xi’an 710082, China.

*** Correspondence:** Xiuhua Liu: liuxh68@chd.edu.cn

# Supplementary Figures and Tables

## Supplementary Figures


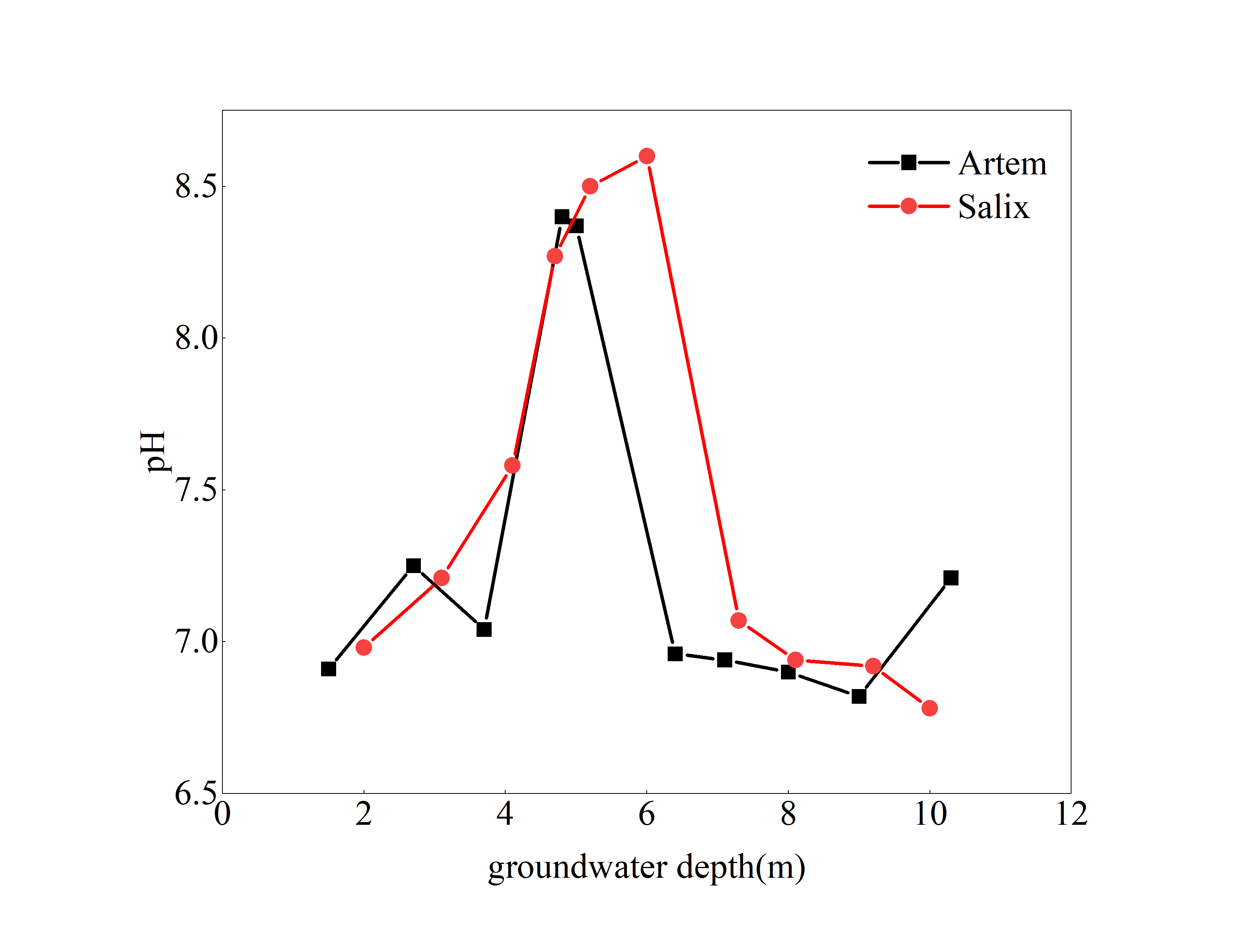


**Supplementary Figure 1.** The varied trend pH of both plants with increasing groundwater depth.


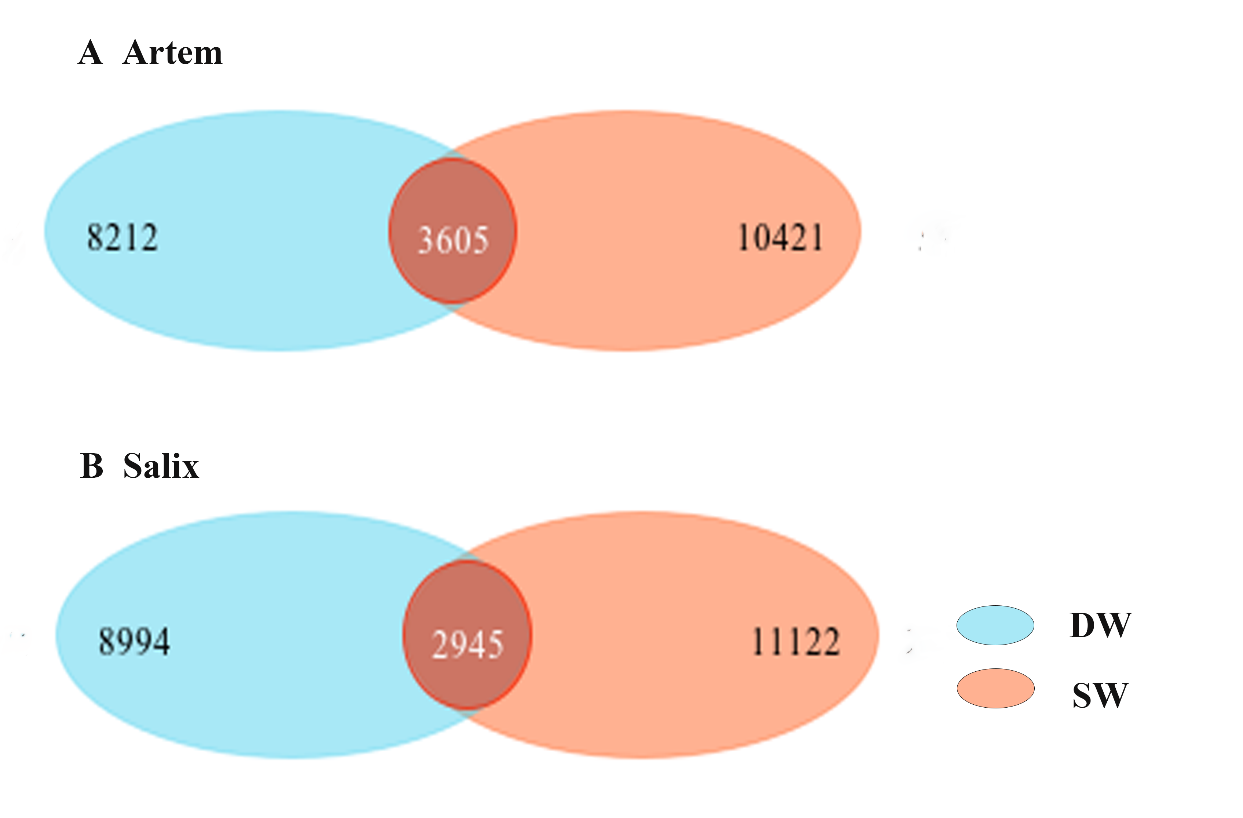


**Supplementary Figure 2.** Venn diagram of soil bacterial communities buried in different groundwater depth.


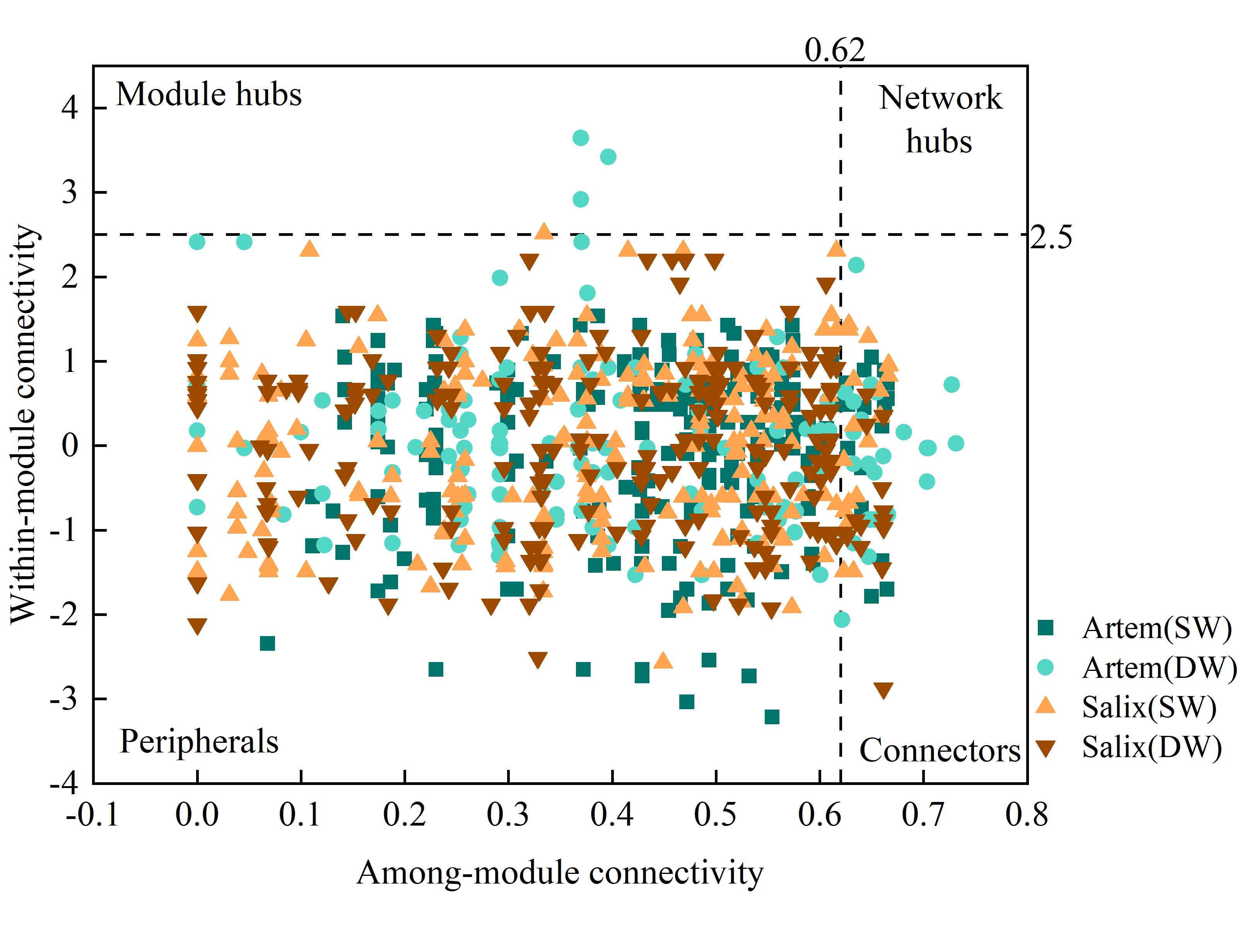


**Supplementary Figure 3.** Distribution rhizospheric bacteria topological structure of two plants under groundwater depth.

## Supplementary Tables

**Supplementary Table 1. Differences of bacterial taxa under different groundwater depth of two plants.**

| bacterial taxa | Artem(SW) | Artem(DW) | Salix(SW) | Salix(DW) |
| --- | --- | --- | --- | --- |
| domain | 2.00±0.00 | 1.80±0.20 | 1.60±0.24 | 1.60±0.24 |
| phylum | 27.80±1.07ab | 28.00±0.84ab | 30.00±0.84a | 27.00±0.95b |
| class | 80.80±1.98a | 82.00±2.30ab | 87.80±1.83b | 78.60±1.99a |
| order | 163.40±3.54ab | 160.00±5.36a | 176.20±3.73b | 154.20±5.68a |
| family | 238.40±5.63ab | 231.40±8.18ab | 251.00±6.19a | 225.00±9.96b |
| genus | 337.20±9.83 | 329.20±17.30 | 335.40±7.59 | 317.00±16.16 |
| species | 51.80±6.11 | 54.00±9.04 | 43.60±4.17 | 50.40±5.10 |

All data are presented as the mean ± standard error. Lowercase letters indicate that the means of bacterial taxa are significantly different (P < 0.05) under different groundwater depth of two plants. *P < 0.05, **P < 0.01, and ***P < 0.001, ANOVA.

**Supplementary Table 2.** **Differences in bacterial phylum under different groundwater depth of two plants.**

| Bacterial phylum | Artem(SW) | Artem(DW) | Salix(SW) | Salix(DW) |
| --- | --- | --- | --- | --- |
| Actinobacteria | 0.398±0.013 | 0.427±0.010 | 0.373±0.032 | 0.441±0.028 |
| Proteobacteria | 0.196±0.013 | 0.196±0.015 | 0.209±0.018 | 0.186±0.021 |
| Chloroflexi | 0.141±0.009ab | 0.125±0.005a | 0.148±0.010ab | 0.16±0.016b |
| Acidobacteria | 0.146±0.005a | 0.118±0.003ab | 0.134±0.014ab | 0.118±0.011b |
| Gemmatimonadetes | 0.028±0.002 | 0.027±0.002 | 0.027±0.002 | 0.025±0.002 |
| Rokubacteria | 0.026±0.005 | 0.024±0.003 | 0.030±0.001 | 0.012±0.002 |
| Firmicutes | 0.016±0.003 | 0.022±0.005 | 0.025±0.008 | 0.011±0.001 |
| Bacteroidetes | 0.01±0.003 | 0.013±0.001 | 0.009±0.001 | 0.010±0.002 |
| Patescibacteria | 0.006±0.001 | 0.007±0.002 | 0.005±0.001 | 0.009±0.002 |
| Planctomycetes | 0.006±0.000 | 0.006±0.000 | 0.006±0.001 | 0.006±0.001 |
| Others | 0.028±0.003 | 0.035±0.003 | 0.033±0.005 | 0.022±0.001 |

All data are presented as the mean ± standard error. Lowercase letters indicate that the means of bacterial phylum are significantly different (P < 0.05) under different groundwater depth of two plants. *P < 0.05, **P < 0.01, and ***P < 0.001, ANOVA.

**Supplementary Table 3. Differences in bacterial class under different groundwater depth of two plants.**

| Bacterial class | Artem(SW) | Artem(DW) | Salix(SW) | Salix(DW) |
| --- | --- | --- | --- | --- |
| Actinobacteria | 0.2±0.02 | 0.2±0.01 | 0.18±0.026 | 0.21±0.024 |
| Thermoleophilia | 0.1±0.008ab | 0.11±0.01ab | 0.09±0.009a | 0.13±0.016b |
| Alphaproteobacteria | 0.1±0.009 | 0.08±0.007 | 0.08±0.006 | 0.1±0.016 |
| Gammaproteobacteria | 0.06±0.004 | 0.08±0.007 | 0.08±0.012 | 0.06±0.011 |
| Subgroup_6 | 0.06±0.002a | 0.05±0.002ab | 0.06±0.005a | 0.04±0.006b |
| MB-A2-108 | 0.04±0.006 | 0.06±0.007 | 0.04±0.008 | 0.04±0.004 |
| KD4-96 | 0.04±0.004 | 0.04±0.002 | 0.05±0.006 | 0.04±0.004 |
| Acidimicrobiia | 0.04±0.004 | 0.04±0.003 | 0.04±0.002 | 0.05±0.002 |
| Blastocatellia_(Subgroup_4) | 0.05±0.007 | 0.03±0.002 | 0.03±0.009 | 0.05±0.01 |
| Chloroflexia | 0.04±0.003a | 0.03±0.001a | 0.03±0.002a | 0.06±0.013b |
| Deltaproteobacteria | 0.04±0.002a | 0.03±0.002b | 0.04±0.004a | 0.03±0.001b |
| Anaerolineae | 0.02±0.002a | 0.02±0.001a | 0.04±0.004b | 0.02±0.003a |
| Gemmatimonadetes | 0.03±0.002 | 0.03±0.002 | 0.03±0.002 | 0.02±0.002 |
| NC10 | 0.03±0.005a | 0.02±0.003a | 0.03±0.001a | 0.01±0.002b |
| Bacilli | 0.01±0.002 | 0.02±0.005 | 0.02±0.009 | 0.01±0.001 |
| Gitt-GS-136 | 0.01±0.001 | 0.01±0.002 | 0.01±0.001 | 0.02±0.004 |
| Subgroup_17 | 0.01±0.001a | 0.01±0.002ab | 0.02±0.002b | 0.01±0.001a |
| Bacteroidia | 0.01±0.003 | 0.01±0 | 0.01±0.001 | 0.01±0.002 |
| TK10 | 0.01±0.001ab | 0.01±0.001ab | 0.01±0.001a | 0.01±0.002b |
| Holophagae | 0.01±0.001 | 0.01±0.001 | 0.01±0.003 | 0.01±0.002 |
| Others | 0.09±0.003 | 0.1±0.005 | 0.1±0.007 | 0.08±0.001 |

All data are presented as the mean ± standard error. Lowercase letters indicate that the means of bacterial class are significantly different (P < 0.05) under different groundwater depth of two plants. *P < 0.05, **P < 0.01, and ***P < 0.001, ANOVA.

**Supplementary Table 4 Topological properties of soil bacterial networks in different groundwater depth (corresponding to Figure7).**

| Topological features | *Artem*(SW) | *Artem*(DW) | *Salix*(SW) | *Salix*(DW) |
| --- | --- | --- | --- | --- |
| Total node | 230 | 132 | 223 | 238 |
| Total links | 9326 | 1961 | 5518 | 6095 |
| Positive links | 4875 | 1032 | 2914 | 3389 |
| Negative links | 4451 | 929 | 2604 | 2706 |
| Average degree | 81.1 | 29.71 | 49.49 | 51.22 |
| Average path length | 1.66 | 1.93 | 1.99 | 2.01 |
| Betweenness centralization | 0.01 | 0.02 | 0.01 | 0.01 |
| Average clustering coefficient | 0.61 | 0.59 | 0.6 | 0.57 |
| Complexity | 40.55 | 12.89 | 24.74 | 25.61 |
| Modularity | 0.34 | 0.43 | 0.41 | 0.38 |
